# Genome-scale reconstruction of metabolic network for a halophilic extremophile, *Chromohalobacter salexigens *DSM 3043

**DOI:** 10.1186/1752-0509-5-12

**Published:** 2011-01-21

**Authors:** Özlem Ates, Ebru Toksoy Oner, Kazim Y Arga

**Affiliations:** 1 Department of Bioengineering, Marmara University, 34722, Istanbul, Turkey

## Abstract

**Background:**

*Chromohalobacter salexigens *(formerly *Halomonas elongata *DSM 3043) is a halophilic extremophile with a very broad salinity range and is used as a model organism to elucidate prokaryotic osmoadaptation due to its strong euryhaline phenotype.

**Results:**

*C. salexigens *DSM 3043's metabolism was reconstructed based on genomic, biochemical and physiological information via a non-automated but iterative process. This manually-curated reconstruction accounts for 584 genes, 1386 reactions, and 1411 metabolites. By using flux balance analysis, the model was extensively validated against literature data on the *C. salexigens *phenotypic features, the transport and use of different substrates for growth as well as against experimental observations on the uptake and accumulation of industrially important organic osmolytes, ectoine, betaine, and its precursor choline, which play important roles in the adaptive response to osmotic stress.

**Conclusions:**

This work presents the first comprehensive genome-scale metabolic model of a halophilic bacterium. Being a useful guide for identification and filling of knowledge gaps, the reconstructed metabolic network *i*OA584 will accelerate the research on halophilic bacteria towards application of systems biology approaches and design of metabolic engineering strategies.

## Background

Extreme environments, generally characterized by abnormal temperature, pH, pressure, salinity, toxicity and radiation levels, are inhabited by various organisms - extremophiles - that are specifically adapted to these particular conditions. Studies on these microorganisms has led to the development of important molecular biology techniques such as polymerase chain reaction (PCR) [1,2] and hence further research has been largely stimulated by the industry's interest on the fact that the survival mechanisms of these microorganisms could be transformed into valuable applications ranging from wastewater treatment to the diagnosis of infectious and genetic diseases [3].

Halophilic microorganisms are extremophiles that are able to survive high osmolarity in hypersaline conditions either by maintenance of high salinity in their cytoplasm or by intracellular accumulation of osmoprotectants such as ectoine and betaine [4]. *C. salexigens *is a halophilic Gammaproteobacterium of the family *Halomonadaceae *with a versatile metabolism allowing not only fast growth on a large variety of simple carbon compounds as its sole carbon and energy source but also resistance to saturated and aromatic hydrocarbons and heavy metals [5,6]. *C. salexigens *with the ability to grow over a wide range of salinities [0.5-4 M NaCl] has been the most euryhaline of the bacteria [7] and to understand the osmoregulatory mechanisms in halophilic bacteria, it has been used as a model organism [5,7-9]. Moreover, *C. salexigens *has also many promising biotechnological applications as a source of compatible solutes, salt-tolerant and recombinant enzymes, biosurfactants and exopolysaccharides [10].

Genome sequence of extremophiles, such as sulphate-reducing archaeon *Archaeaglobus fulgidus *[11], halophilic archaeon *Halobacterium *species NRC-1 [12] and acidophilic bacterium *Acidithiobacillus ferrooxidans *[13] have been reported earlier. Since the publication of the genome of *C. salexigens *DSM 3043 [14] the biological knowledge about this strain has significantly increased and various methods that allow the genomic analysis and genetic manipulation have been developed [15,16]. On the other hand, systematic analysis of its metabolic and biotechnological capacities have not been performed yet. This is, at some level, due to the lack of an *in silico *comprehensive metabolic model that enables the integration of canonical experimental data in a coherent fashion.

Metabolic reconstruction is non-automated and iterative decision-making process through which the genes, enzymes, reactions and metabolites that participate in the metabolic activity of a biological system are identified, categorized and interconnected to form a network [17]. The reconstruction process has been reviewed conceptually in literature [17-22] and, recently, a standard operating protocol giving a detailed overview of the necessary data and steps has been published [23]. To date, genome-scale metabolic reconstructions for more than 50 organisms have been published and this number is expected to increase rapidly. Therefore, the need for developing automated, or at least semi-automated, ways to reconstruct metabolic networks is growing. A limited number of software tools, such as Pathway tools [24], metaSHARK [25], Simpheny (Genomatica), which aim at assisting and facilitating the reconstruction process are available. However, recent reviews [18,26] highlight current problems with genome annotations and databases, which make automated reconstructions challenging and thus they require manual evaluation. Genome-scale metabolic reconstructions have been successfully applied to several organisms across eukaryotic (e.g., *Saccharomyces cerevisiae *[21,27-29], human [30], *Arabidopsis thaliana *[31]), prokaryotic (e.g., *Escherichia coli *[32-34], *Bacillus subtilis *[35], *Helicobacter pylori *[36,37], *Lactococcus lactis *[38], *Staphylococcus aureus *[39,40], *Clostridium acetobutylicum *[41], *Pseudomonas putida *[42], *Pseudomonas aeruginosa *[43], *Geobacter metallireducens *[44], *Corynebacterium glutamicum *[45]), and archaeal (e.g., *Methansoarcina barkeri *[46], *Halobacterium salinarum *[47] species). Being a useful guide for identification and filling of knowledge gaps, these metabolic networks have been used toward simulation of the cellular behavior under different genetic and physiological conditions, contextualization of high-throughput data, directing hypothesis driven discovery, interrogation of multi-species relationships and topological analysis (See [17] for an extensive review).

Here, a genome-scale reconstruction of *C. salexigens *DSM 3043's metabolism was established based on genomic, biochemical and physiological information. Being the first comprehensive metabolic model of a halophilic bacterium, it was labeled as *i*OA584 following the naming convention proposed by [33]. The predictive potential of the model was validated not only against literature data on the *in vivo C. salexigens *phenotypic features, the transport and use of different substrates but also against experimental observations on the choline - betaine and ectoine synthesis pathways which are important parts of the osmoadaptation mechanism.

## Methods

### Genome Annotation

The complete genome sequence of *C. salexigens *DSM 3043 has been assembled in 2005 by the Joint Genome Institute [14] and gene annotations are available online at the web-sites of Computational Biology at ORNL [48] and Joint Genome Institute [14], which represent computational platforms enabling the corresponding enzymes in addition to gene catalog. *C. salexigens *DSM 3043 genome size is 3.696 Mb with 3352 candidate protein-encoding gene models.

### Reconstruction Process

For the reconstruction of a genome-scale metabolic network of the halophilic bacterium *C. salexigens *DSM 3043, a non-automated but iterative decision-making process is designed based on the conceptual reviews [18,19,22] and published protocol [23]. In the first stage, a draft reconstruction was built from gene-annotation data [48,49] coupled with information from online databases, which link genes to functional categories and help bridge the genotype-phenotype gap. For the association of the enzymes to the biochemical reactions, biochemical information databases KEGG [50], BiGG [51], ExPASy [52], BioCyc [53] and BRENDA [54], which provide comprehensive information on enzymes and biochemical reactions, were employed to extract metabolic reactions, their stoichiometry and thermodynamic constraints (i.e. reversibility). As a result of the first stage, an initial catalog of gene-enzyme-reaction associations was prepared. In the second stage, the draft reconstruction was refined semi-automatically through gap analysis. Using the draft catalog, the stoichiometric matrix, the reaction and metabolite adjacency matrices [55] were constructed, metabolic maps were drawn and topological analysis [56,57] was performed. Analysis of the preliminary version of the network indicated the occurrence of metabolites not connected with the overall metabolic network, i.e. the presence of dead-end metabolites. The resulting shortage was overcome mostly by manually searching biochemical information databases [50-54] and carrying out a comprehensive literature survey on metabolisms of *C. salexigens *[5-9,16,58,59]. In the last stage, the biomass formation and transport reactions, which describe the intra- and extracellular exchange of metabolites, were added to the metabolic network predominantly based on the experimental evidence on phenotypic characterization of the strain [5-9,16,58,59]. The reconstructed metabolic network was automatically converted into a mathematical model that could be analyzed through constraint-based approaches, and was validated through comparison of model predictions with phenotypic data.

### Constraint-based Modeling

The interconnectivity of metabolites in a biochemical reaction network can be represented by a set of equations defining the stoichiometric conversion of substrates into products [60]. The reconstructed metabolic network was represented by a stoichiometric matrix, S (*m *× *n*) where *m *is the number of metabolites and *n *is the number of reactions. The corresponding entry in the stoichiometric matrix, S_*ij*_, represents the stoichiometric coefficient for the participation of the i^th ^metabolite in the j^th ^reaction. A constraint-based optimization framework, Flux Balance Analysis (FBA) [61,62], was then recruited to solve the linear programming problem under steady-state criteria represented by the equation () where v is a vector of reaction fluxes. Since the optimization problem belongs to an under-determined system, there exist multiple solutions. To find a particular solution for reaction fluxes, the cellular objective of producing the maximum amount of biomass constituents was optimized [63]. The employment of optimal growth assumption has allowed successful calculation of phenotypic behaviour in FBA of reconstructed metabolic models of several microorganisms [34-36,38,40-42,46,47], suggesting that their metabolic networks have evolved for the optimization of the specific growth rate under several carbon source limiting conditions. Constraints need to be imposed on the system in the form of inequality () where α and β are the lower and upper limits placed on each reaction flux, respectively. The constraint-based optimization problem was solved using MATLAB 7.4 (The Mathworks, Inc.).

### Biomass Formulation

No thorough biomass composition has been published for *C. salexigens*. The use of a generic biomass formation reaction in FBA simulations was previously tried and led to successful predictions [34,39,64]. Hence, based on the experimental evidences on genome similarity [7], phylogenetic classification and results from the comparative analysis of the *C. salexigens *metabolic network with other published reconstructed networks [27,34,35,39,42,43,46,47], the relative production of metabolites required for growth was taken from the published composition of *E. coli i*AF1260 [34].

### Flux Variability Analysis

The flux variabily analysis was performed [65] to observe the alternate optimal flux distributions. Briefly, the optimal value of the objective function was calculated by FBA simulation; then, with the objective function fixed at the optimal value, for each reaction the maximum and minimum possible fluxes were computed. The two values calculated for each reaction characterize its variability.

## Results And Discussion

### Metabolic Reconstruction Process

Based on the conceptual reviews [19,18,22] and published protocol [23], a non-automated but iterative three-stage process was designed to reconstruct a genome-scale metabolic network of the halophilic bacterium *C. salexigens *DSM 3043.

In the first stage, a draft catalog of gene-enzyme-reaction associations was prepared via coupling genome annotation data [48,49] with biochemical information databases [50-54]. The genome annotation resources for *C. salexigens *[48,49] not only include genetic information such as genome position, coding region, locus tag, gene product function, but also represent assignments of gene products to PRIAM categories, COG functional groups, KEGG orthologies and pathways, and Enzyme Commission (EC) numbers. All these information were assembled and analyzed manually to identify candidate metabolic functions. In the first step, the pathway databases, namely KEGG [50] and BiGG [51], were systematically searched for the associations of the metabolic reactions to the enzymes. At this step, KEGG pathway assignments and EC numbers, which represent a hierarchical classification of enzymatic reactions and are commonly utilized as identifiers of enzymes in the analysis of complete genomes, played important role in bridging the genomic repertoire of gene models to the chemical repertoire of metabolic pathways. However, several EC numbers were assigned to signaling or regulatory proteins, whose functions are not normally considered in metabolic reconstructions. For instance, Csal2070 gene was assigned for a repressor protein LexI (EC 3.4.21.88) functioning in SOS regulation. Therefore, these assignments were carefully checked and not included in the draft reconstruction. Another important point to be emphasized is the incompleteness of pathway databases. Although very high percentages (66.6%) of the enzymes were associated with the reactions, there were missing reactions that were not represented in these databases. In the second step, enzyme information databases, namely ExPASy [52], BioCyc [53] and BRENDA [54] were explored to include the missing reactions to the model. Since EC numbers were known from previously obtained gene-annotation data, enzymes could be connected with accurate metabolic reactions. For example, the reactions for carbonyl reductase (EC 1.1.1.184), malate synthase (EC 2.3.3.9) and creatinase (EC 3.5.3.3) were obtained from ExPASy, BRENDA and BioCyc databases, respectively. The outcome of the first stage was an initial catalog of gene-enzyme-reaction associations.

Second stage comprised of semi-automatically refinements of the draft reconstruction through gap analysis. Using the draft catalog of gene-enzyme-reaction associations, the stoichiometric matrix, the reaction and metabolite adjacency matrices were constructed, metabolic maps were drawn and topological analysis was performed [55,56] Analysis of the preliminary version of the network indicated the occurance of metabolites not connected with the overall metabolic network, i.e. the presence of dead-end metabolites. Their presence might be due to a misassignment of a gene function or to missing reactions linking these metabolites with the overall network. The resulting shortage was overcome mostly by manually searching other biochemical information databases, namely ExPASy [52], BioCyc [53] and BRENDA [54]. In addition for these enzyme-reaction associations, the required information was obtained from literature. For instance, in the utilization pathway of tagatose, tagatose-6-phosphate kinase reaction (EC 2.7.1.144) was present in the model; but, an essential intermediate step, i.e. the formation reaction of tagatose 6-phosphate from tagatose, was missing in the model. Subsequently, tagatose kinase reaction (EC 2.7.1.101) was included to the model. In some cases, gap analysis indicated the lack of numerous steps in several pathways. For example, in arabinose metabolism 5 additional metabolic reactions (EC 1.1.1.46, EC 3.1.1.15, EC 4.2.1.25, EC 4.2.1.43 and EC 1.2.1.26) were required to link dead-end metabolites to the metabolic model. At this stage, stoichiometrically unbalanced reactions were also checked. Normally, there are two common errors causing unbalanced reactions [23]: Missing proton and/or water, or when the stoichiometric coefficient of at least one metabolite is wrong. All the metabolic reactions were tested for mass and charge balancing and several reactions required corrections. For example, in the reaction catalyzed by glucokinase (EC 2.7.1.2, Csal0935), which was obtained from KEGG [50], a proton was missing.

In the last stage, the reconstructed metabolic network was automatically converted into a mathematical model that could be analyzed through constraint-based approaches, and was validated through comparison of model predictions with phenotypic data. The biomass formation and transport reactions, which describe the intra- and extracellular exchange of metabolites, were added to the metabolic network predominantly based on the experimental evidence [5-9,16,58,59] on phenotypic characterization of the strain and then FBA simulations on various carbon sources were performed to verify the model. For example, uptake of macro nutrients (e.g., amino acids, sucrose, glucose), secretion of by-products (e.g., lactate, ammonia, betaine), and exchange of free compounds (water, carbon dioxide, oxygen) were added since they represent essential cellular inputs and outputs. The metabolic model was updated iteratively using the above procedure until the *in silico *phenotypic characterizations were completely represented by the simulation results.

### Characteristics of the Reconstructed Metabolic Network of *C. salexigens*

The reconstruction process resulted in a metabolic network that consisted of 1387 metabolic reactions including biomass reaction and 1411 metabolites (Additional File 1). The model is composed of 876 enzymatic reactions, 510 transport reactions; 920 intracellular and 491 extracellular metabolites and throughout the reconstruction process, 584 protein-encoding gene models have been assigned to the metabolic reactions (Table 1). For 97.7% of all enzymatic reactions, a corresponding gene-enzyme-reaction association has been assigned in the model.

**Table 1:** Network characteristics of the reconstructed metabolic network of *C. salexigens*

Protein-encoding gene models	584
Metabolites	1411
Intracellular metabolites	920
Extracellular metabolites	491
Reactions	1386
Enzymatic reactions	876
Transport fluxes	510
Reactions with protein-encoding gene model assignments	886
Enzymatic reactions	856
Transport fluxes	30

A large amount of enzymes, which were included by the metabolic model, were monofunctional (80.65%) whereas the rest were multifunctional accepting several different substrates. Therefore, the published genome for their corresponding genes were carefully checked during reconstruction process in order not to lead to false gene-enzyme-reaction associations in the reconstructed genome-scale metabolic model.

**Figure 1:**
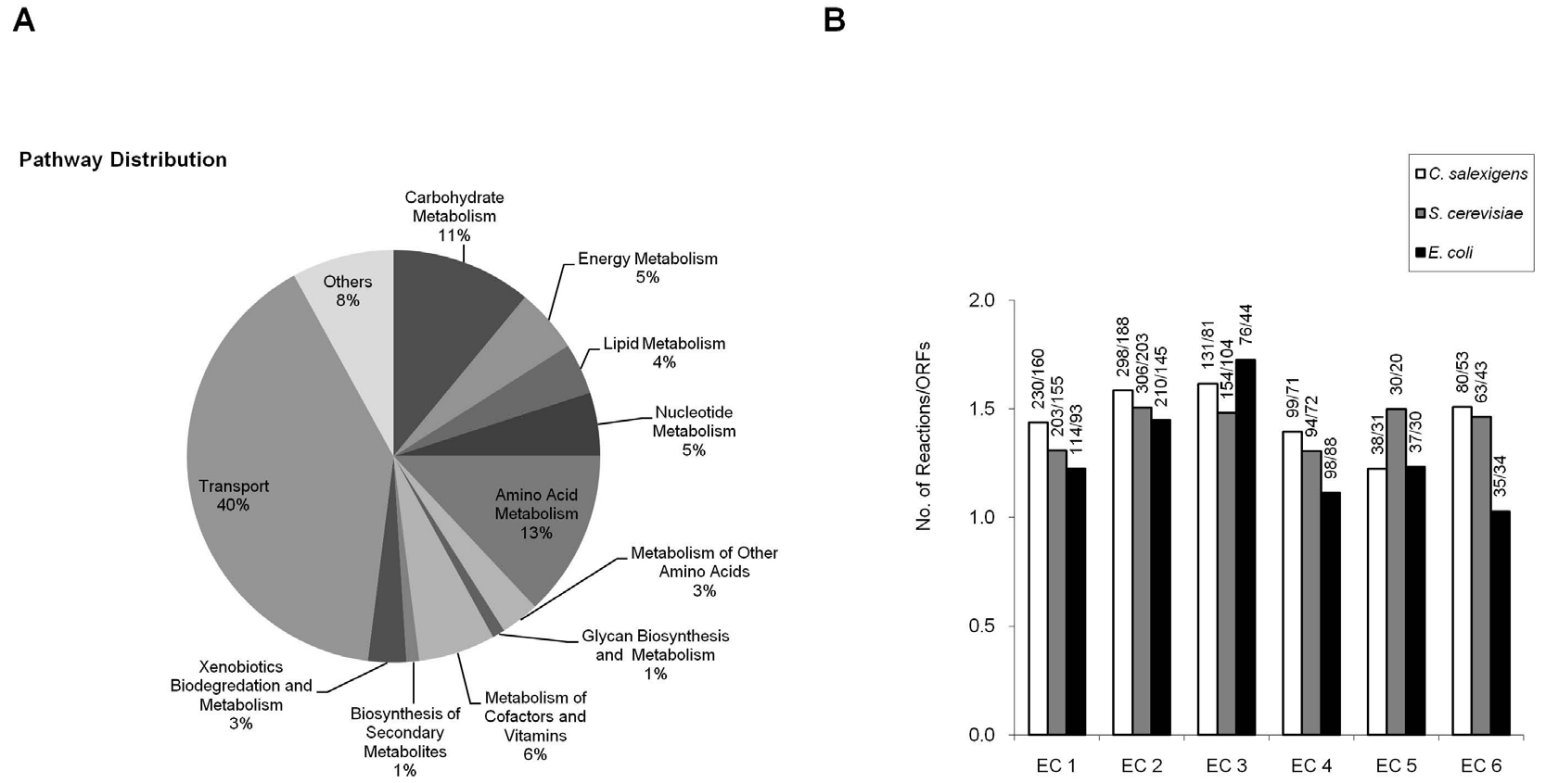
**Characteristics of the reconstructed metabolic network *i*OA584**. **A**: Distribution of the 12 main metabolic pathways in *i*OA584. **B**: Comparison of the distribution of enzyme classes in *C. salexigens, E. coli *and *S. cerevisiae*.

The enzymes included in *i*OA584 were divided into 12 main categories based on their functional roles (Figure 1A). The transport category was found to be the subsystem with the highest number of enzymes (40%), highlighting the importance of cellular transport for *C. salexigens*. Most of the transport reactions were included into the network based on physiological data and the abundance of transport reactions agrees well with the experimental findings that this organism has an excellent adaptation to osmotic stress [8] and is able to utilize various carbon sources as sole energy source. However, the high number of transport reactions with no gene assignment (94% of transport reactions) in *C. salexigens *points to the fact that further work is needed to characterize the mechanisms and genetic machinery involved in the transport of molecules in halophilic bacteria. For example, although the halophilic bacterium is known to be able to utilize various carbon sources as sole energy source, only 4 genes (Csal0010, Csal1144, Csal0500, and Csal1728) were associated with sugar transport mechanisms in the annotated genome of *C. salexigens *[38]. In addition, only one of them (Csal0010) has been associated with an enzyme (EC.2.7.8.20) in BioCyc [53]. Deciphering the transport phenomena in halophilic bacteria is an important issue, since understanding the osmoprotectant uptake mechanisms in natural environments is a key point in achieving an efficient osmoadaptation. Therefore, for further studies, a detailed biophysical classification of the 342 candidate gene models related to the transport mechanism was presented (See Additional File 2 for the complete list of genes and their annotations).

Moreover, *C. salexigens *is known for its capability to utilize many amino acids as a carbon and nitrogen source [5,6,59]. The presence of high number of enzymes (13%) in the amino acid metabolism is in agreement with the fact that the *de novo *synthesis pathways for all 20 amino acids are present in *C. salexigens*' genome [14,48]. To validate *in silico *amino acid utilization as a carbon and nitrogen source, FBA simulations were carried out and growth on all of the 20 amino acids were obtained. For instance, at a specific uptake rate (1 mmol/gDW/h) of isoleucine, growth rate was calculated as 0.129 h^-1^. Additionally, glycan biosynthesis and metabolism; and biosynthesis of secondary metabolites have the lowest number of enzymes (1%).

Throughout the reconstruction process, 584 protein-encoding gene models have been assigned to the metabolic reactions. The distribution of the ratios of number of reactions per number of gene models in each enzyme class [27,32] was investigated in the reconstructed model *i*OA584 (Figure 1B). In the metabolic network of *C. salexigens*, hydrolases (EC 3) were positioned primarily, followed by transferases (EC 2), ligases (EC 6), oxidoreductases (EC 1), lyases (EC 4), and isomerases (EC 5). Hence ligases and transferases were less substrate specific than the other enzyme classes in *C. salexigens*, as in the case of *E. coli *[32], whereas in *S. cerevisiae *isomerases and transferases were found to be the least substrate-specific enzyme classes [27].

Related species of the same domain share a substantial amount of conserved reactions for essential biological processes [66-68]. The metabolic network *i*OA584 was also compared with previous metabolic models from different domains [27,34,35,39,42,43,46,47] to identify the conserved reactions in *i*OA584. As expected, highest number of metabolic reactions were shared by *E. coli *(*i*AF1260) with 320 reactions, *P. aeruginosa *(*i*MO1056) with 309 reactions and *P. putida *(*i*JN746) with 282 reactions. Number of shared metabolic reactions for *S. cerevisiae*, *S. aureus *N315, *B. subtilis*, and *H.salinarium *were obtained as 274, 265, 260, and 221, respectively; while *C. salexigens *and *M. barkeri *association indicated the lowest number with 205 metabolic reactions.

**Figure 2:**
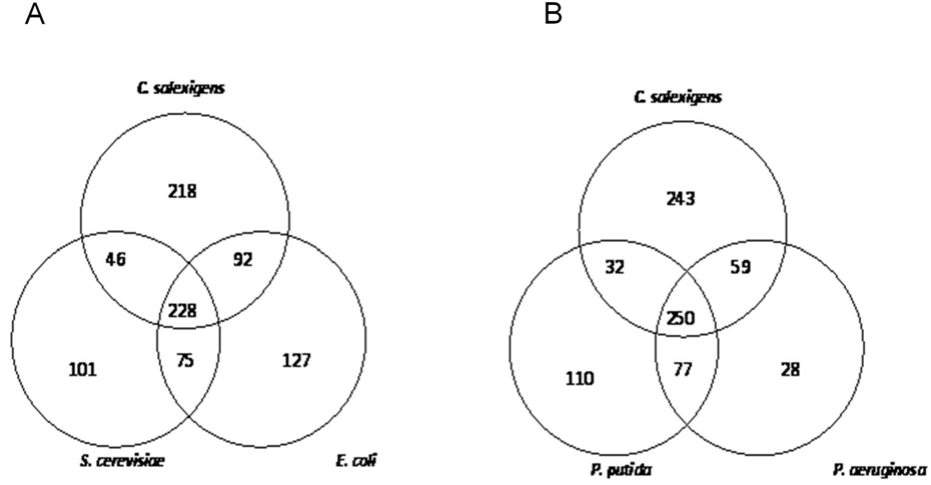
**Comparison of the reconstructed metabolic network with previous metabolic models from different domains**. **A**: The distribution of reactions in *C. salexigens, E. coli and S. cerevisae ***B**: The distribution of reactions in *C. salexigens,P. putida *and *P.aeruginosa*

The distribution of the reactions for *C. salexigens*, *E. coli *and the eukaryote *S. cerevisiae *(Figure 2A) indicated an interior set of 228 reactions in all of the three metabolic models with the following pathway distribution; 95 from amino acid, 53 from carbohydrate, 52 from metabolism of cofactors and vitamins, 43 from nucleotide, 27 from energy and 18 from lipid metabolism. A number of reactions were found to be involved in more than one pathway such as reactions catalyzed by alcohol dehydrogenase (ADH) enzymes (EC 1.1.1.1) that can be found in carbohydrate, lipid and amino acid metabolisms in agreement with literature [69]. 37.3% of the total reactions were unique to *C. salexigens i*OA584 most of which were from amino acid (38 reactions) metabolism followed by carbohydrate metabolism (31 reactions). Comparison of the distribution of metabolic reactions for *C. salexigens*, *P. putida *and *P. aeruginosa *(Figure 2B) showed a higher interior set with 250 reactions as expected, since species of the same domain share a substantial amount of conserved reactions for essential biological processes [68]. A similiar pathway distribution was observed whereas most of the reactions were involved in amino acid, carbohydrate metabolism and lipid metabolism with 118, 89 and 57 reactions, respectively.

### Capabilities of the metabolic network - Phenotypic characterization *in silico*

One of the major requirements for a reconstructed network is its compatibility with the physiology of the organism which in turn is highly essential when using the model in understanding the diverse mechanisms of the organism. In the present study, *in silico *phenotypic characterization constitutes an essential step of the reconstruction process. At the last stage of the reconstruction process, FBA simulations were performed with various growth media to test for incapabilities of the model in representing the phenotypic features in literature [5-9,16,58,59] (Table 2). For example, the metabolic model *i*OA584 was not able to utilize galactitol, tagatose, xylose, erythritol, arabinose, malonate, propionate and glycerate due to the absence of several exchange and enzymatic reactions. These shortages were resolved via manual searching of biochemical information databases [52-54] and by addition of 13 reactions (EC 1.1.1.16, EC 1.1.1.175, EC 1.1.1.46, EC 1.2.1.15, EC 1.2.1.26, EC 3.1.1.15, EC 2.7.1.101, EC 2.7.1.27, EC 2.7.2.15, EC 4.2.1.25, EC 4.2.1.43, rxn978 and rxn1314) into the network. The metabolic network was updated until the complete *in silico *phenotypic characterization was achieved.

**Table 2:** *In silico *predictions of the phenotypic features.

*Phenotype*	*(in vivo/in silico)*	*Related Enzymes*	*Related Reactions*
**Catalase activity**	(+/+)	Catalase (1.11.1.6)	rxn87
**Citrate activity**	(+/+)	citrate synthase (2.3.3.1)	rxn328
**Urease activity**	(+/+)	Urease (3.5.1.5)	rxn610
**Nitrate reduction**	(+/+)	Nitrate reductase (1.7.99.4)	rxn216-rxn218

***Substrate***	***(in vivo/in silico)***	***Transport Reactions***	***Utilization Reactions***

**Acetate**	(+/+)	rxn964	rxn527, rxn559, rxn817
**Adonitol**	(-/-)	-	-
**Glycine Betaine**	(+/+)	rxn1112, rxn1113	rxn136, rxn137, rxn243
**Butyrate**	(-/-)	-	-
**Caprylate**	(-/-)	-	-
**Cellobiose**	(-/-)	-	-
**Choline**	(+/+)	rxn1004, rxn1005	rxn83, rxn107, rxn580
**Citrate**	(+/+)	rxn1007	rxn328, rxn702, rxn726, rxn727
**Creatine**	(-/-)	-	-
**D-fructose**	(+/+)	rxn1079, rxn1080	rxn62, rxn337, rxn433, rxn781
**D-galactose**	(+/+)	rxn1091	rxn53
**D-glucose**	(+/+)	rxn877, rxn881, rxn882	rxn82, rxn422
**DL-glycerate**	(+/+)	rxn1114	rxn64
**DL-α-aminobutyrate**	(-/-)	-	-
**D-mannitol**	(+/+)	rxn1173	rxn33, rxn62
**D-mannose**	(+/+)	rxn1161	rxn33, rxn781
**D-melibiose**	(-/-)	-	-
**D-raffinose**	(-/-)	-	-
**D-ribose**	(+/+)	rxn1274	rxn418
**D-sorbitol**	(+/+)	rxn1284	rxn17
**L-tartrate**	(+/+)	rxn1312	rxn729
**D-trehalose**	(+/+)	rxn1327, rxn1328	rxn562
**Dulcitol (galactitol)**	(+/+)	rxn1101	rxn53
**D-xylose**	(+/+)	rxn1385	rxn22
**Erythritol**	(+/+)	rxn1060	rxn427
**Ethanol**	(+/+)	rxn1065	rxn2
**Fumarate**	(+/+)	rxn1081	rxn155, rxn156, rxn158 - rxn162, rxn720, rxn753 - rxn755
**Galactosamine**	(-/-)	-	-
**Gluconolactone**	(-/-)	-	-
**Glutamate**	(+/+)	rxn1109	rxn763, rxn854
**Glycerol**	(+/+)	rxn1116	rxn1, rxn429
**Glycine**	(+/+)	rxn1118	rxn182, rxn183, rxn257, rxn258, rxn605, rxn695, rxn802, rxn848, rxn859
**Inulin**	(-/-)	-	-
**Lactate**	(-/-)	-	-
**L-alanine**	(-/-)	-	-
**L-arabinose**	(+/+)	rxn978	rxn52
**L-arginine**	(+/+)	rxn979	rxn275, rxn616, rxn661, rxn753, rxn807
**L-asparagine**	(+/+)	rxn981	rxn597
**L-fucose**	(-/-)	-	-
**L-glutamine**	(+/+)	rxn1103	rxn175, rxn184, rxn389, rxn699, rxn806, rxn863, rxn868 - rxn872
**L-lysine**	(+/+)	rxn1153	rxn104, rxn814
**L-methionine**	(-/-)	-	-
**L-ornithine**	(+/+)	rxn1209	rxn267, rxn268, rxn750
**L-proline**	(+/+)	rxn1248	rxn195, rxn803
**L-rhamnose**	(-/-)	-	-
**L-serine**	(+/+)	rxn1290	rxn37, rxn256, rxn257, rxn297, rxn298, rxn514 - rxn520, rxn721, rxn722, rxn751, rxn799, rxn800
**L-threonine**	(-/-)	-	-
**L-valine**	(-/-)	-	-
**Malate**	(+/+)	rxn1155	rxn46, rxn47, rxn84, rxn85, rxn720
**Malonate**	(+/+)	rxn1156	rxn122
**Maltose**	(+/+)	rxn1157	rxn587
**Meso-inositol**	(+/+)	rxn1170	rxn96
**Oxalate**	(-/-)	-	-
**Propionate**	(+/+)	rxn1249	rxn433
**Putrescine**	(-/-)	-	-
**Sarcosine**	(-/-)	-	-
**Succinate**	(+/+)	rxn1305	rxn97, rxn98, rxn156, rxn159, rxn160, rxn163, rxn700, rxn841
**Sucrose**	(+/+)	rxn883, rxn884, rxn885	rxn337
**Tagatose**	(+/+)	rxn1314	rxn39, rxn439
**α-lactose**	(-/-)	-	-

The resultant metabolic model *i*OA584 has the ability to verify reported *C. salexigens *phenotypic features [5-9,16,58,59] through *in silico *FBA simulations. *C.salexigens *is able to grow aerobically and has ability for anaerobic respiration with nitrate. This microorganism is catalase and citrate positive, oxidase negative. Nitrate can be reduced to nitrite in contrast nitrite cannot be reduced [5,6,59]. The *in silico *aerobic and anerobic growth simulations were performed with biomass as the objective function at a specific glucose uptake rate of 3 mmol/gDW/h and for anaerobic respiration with 1 mmol/gDW/h nitrate as an electron acceptor instead of O_2_. The growth rates were determined as 0.1934 h^-1 ^and 0.0645 h^-1 ^for aerobic and anaerobic conditions, respectively. As such, catalase, citrate, urease activities and nitrate reduction simulations were also consistent with literature data (Table 2). Acetoin, indole, lysine decarboxylase, ornithine decarboxylase and phenylalanine deaminase could not be produced by *C. salexigens i*OA584 as also reported *in vivo *[6]. Literature data on the transport and use of 59 different substrates were also verified *in silico *by fixing the externally transport reaction of fluxes (3-10 mmol/gDW/h) and investigating the associated utilization reaction fluxes and objective function biomass flux to assess a positive growth. For example, 1 mmol/gDW/h uptakes of fructose and sucrose resulted in growth rates of 0.1290 h^-1 ^and 0.0646 h^-1^, respectively.

**Figure 3:**
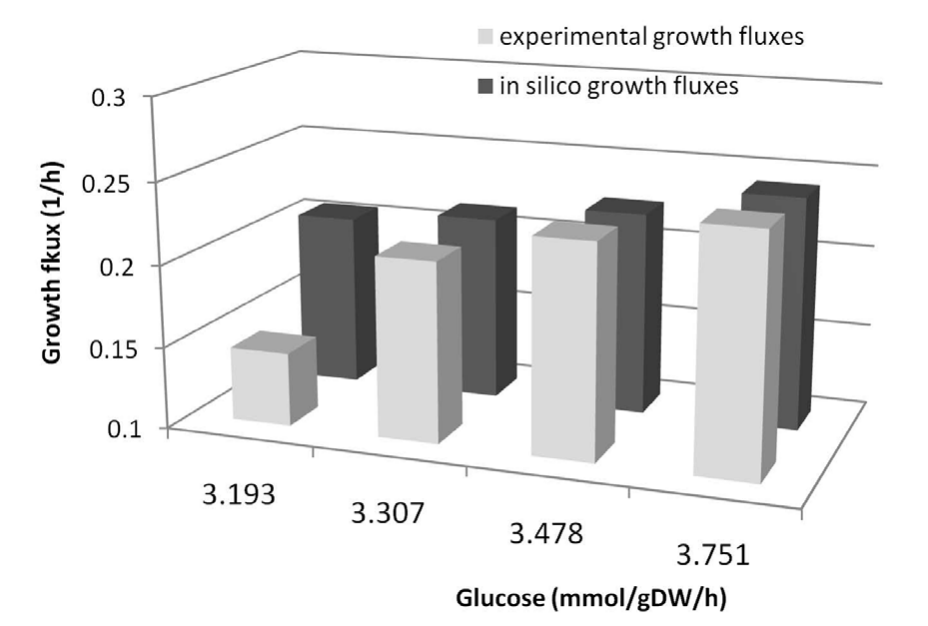
Experimental and *in silico *growth fluxes for batch cultivation of *C.salexigens *when glucose uptake rate was varying between 3.193 - 3.751 mmol/gDW/h

Additionally, the FBA simulations were performed in order to validate experimental growth rate values with glucose as the only carbon source in chemically defined media which were reported [70]. Experimental and *in silico *growth fluxes for batch cultivation of *C. salexigens *at varying glucose uptake rates ( 3.193 - 3.751 mmol/gDW/h) were illustrated in Figure 3. Whereas a higher growth rate was predicted for 3.193 mmol/gDW/h, simulations were in significant agreement with the experimental data with as low as 1.5 to 2.5% errors for the other glucose concentrations (3.307, 3.478 and 3.751 mmol/gDW/h).

Since the flux distribution of overall network map might be useful in investigating and improving FBA analysis, Omics Viewers Tool of BioCyc [53] was used to illustrate *in silico *flux distribution in *C. salexigens *metabolic pathways. Reaction flux data and gene information were provided for Omics Viewer to generate overall diagram colorized with flux data. The details of the connectivity aspects of the reconstructed metabolic network (Additional File 3), the overall map of the reconstructed network and its detailed batch images obtained were also supplemented (Additional File 4).

### Case study on osmoadaptation

Generally, halophiles can adapt to the saline environment by either intracellular accumulation of salts, or exclusion of salts and production or accumulation of different classes of organic solutes (osmoprotectants) [71,72]. *C. salexigens *has been used comprehensively as a model organism in osmoadaptation studies due to its ability to grow over a wide range of salinities [6-8]. Osmoadaptation in *C. salexigens *is mainly achieved by *de novo *synthesis of two compatible solutes, namely ectoine and hydroxyectoine, which are of industrial and biological interest due to their biostabilizing properties. In addition, when these solutes are provided externally, *C. salexigens *accumulates other osmoprotectants such as choline and glycine betaine. Besides the betaine exchange that is common in bacteria, the rarely encountered betaine biosynthesis pathway from choline has been characterized in *C. salexigens *to some extend at the biochemical level [5,8,59,73,74]. Further research on the genes and metabolic pathways responsible for the biosynthesis of compatible solutes will not only find numerous applications in biomedicine, agriculture, food and fermentation industries but also expand our knowledge on the prokaryotic adaptation mechanisms to abiotic stresses like high salinity [72].

Via integration of data from *in vitro *metabolic and genetic analyses, in further studies, the presented genome scale model iOA584 could be used to elucidate osmoadaptation mechanisms and to design strategies (i.e. optimizing culture media, genetical engineering of the microorganism) for optimum production of compatible solutes such as ectoine, which has industrial applications for cosmetics and dermopharmacy and is widely used in stabilizing enzymes for molecular biology.

Here, *C. salexigens i*OA584 was used to simulate the experimental observations on osmoadaptation of *C. salexigens*, in order to demonstrate that the model could be used for further studies in understanding the metabolic pathways behind osmoadaptation and to design or improve the adaptation mechanisms in extromophiles.

**Figure 4:**
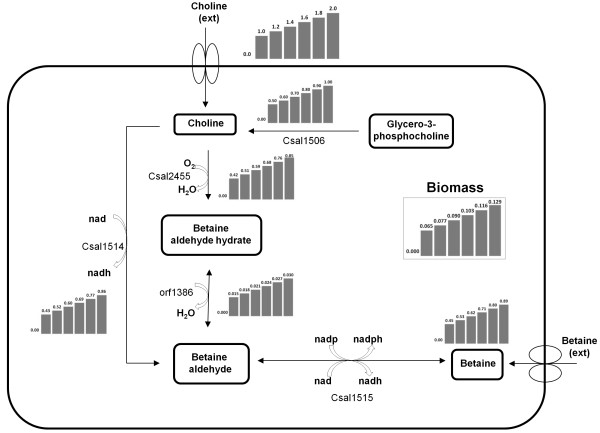
***In silico *model simulations of the choline - betaine pathway of the osmoadaptation mechanism**. In *C. salexigens*, the osmoprotectant betaine is synthesized from its precursor choline in two steps. Whereas the first step from choline to betaine aldehyde is catalyzed either by membrane-bound choline dehydrogenase (EC 1.1.99.1, CsaI1514) or by a ferredoxin-dependent choline monooxygenase (EC 1.14.15.7, CsaI2455), betaine aldehyde dehydrogenase (EC 1.2.1.8, CsaI1515) is involved in the second, betaine aldehyde to betaine step. FBA simulations were performed with biomass as the objective function and 3 mmol/gDW/h glucose uptake rate and the computed metabolic flux values in mmol/gDW/h are shown on bar charts. Uptake of exogeneous choline was restricted to 0, 1.0, 1.2, 1.4, 1.6, 1.8 and 2 mmol/gDW/h.

In *C. salexigens*, the osmoprotectant betaine is synthesized from its precursor choline in two steps (Figure 4). In the first step, choline is converted into betaine aldehyde by membrane-bound choline dehydrogenase (EC 1.1.99.1, Csal1514) or by a ferredoxin-dependent choline monooxygenase (EC 1.14.15.7, Csal2455). Then, betaine aldehyde dehydrogenase (EC 1.2.1.8, CsaI1515) catalyzes the conversion of betaine aldehyde to betaine in the second step. Previously, Canovas and coworkers (1998) have investigated the transport of choline and its conversion to the osmoprotectant compound glycine betaine in *C. salexigens*. They reported that the growth of *C. salexigens *(with glucose as the sole carbon source) was stimulated by the presence of choline and that the presence of betaine had an inhibitory effect on the intracellular oxidation of choline.

For validation of the model's predictive potential, *in silico *model simulations of the choline - betaine pathway of the osmoadaptation mechanism were compared with these experimental observations [5,7-9,59]. FBA simulations were performed with biomass as the objective function and 1 to 3 mmol/gDW/h glucose uptake rate (Figure 4). Via restriction of uptake of exogeneous choline to various values between 1 to 2 mmol/gDW/h, monotonic increase in the biomass flux (from 0.065 to 0.129 h^-1^) and in betaine production flux (from 0.45 to 0.89 mmol/gDW/h) were observed; hence stimulation of growth by the presence of choline was predicted, which is in agreement with the reported experimental observations [58]. It is known that the resulting solution of FBA especially when applied to genome scale models is normally not unique [65]. Therefore, the flux variability analysis was performed to observe the alternate optimal flux distributions in FBA simulations. Results showed that the fluxes are in general not affected since the range of variabilities for each flux were lower than 0.1%.

**Figure 5:**
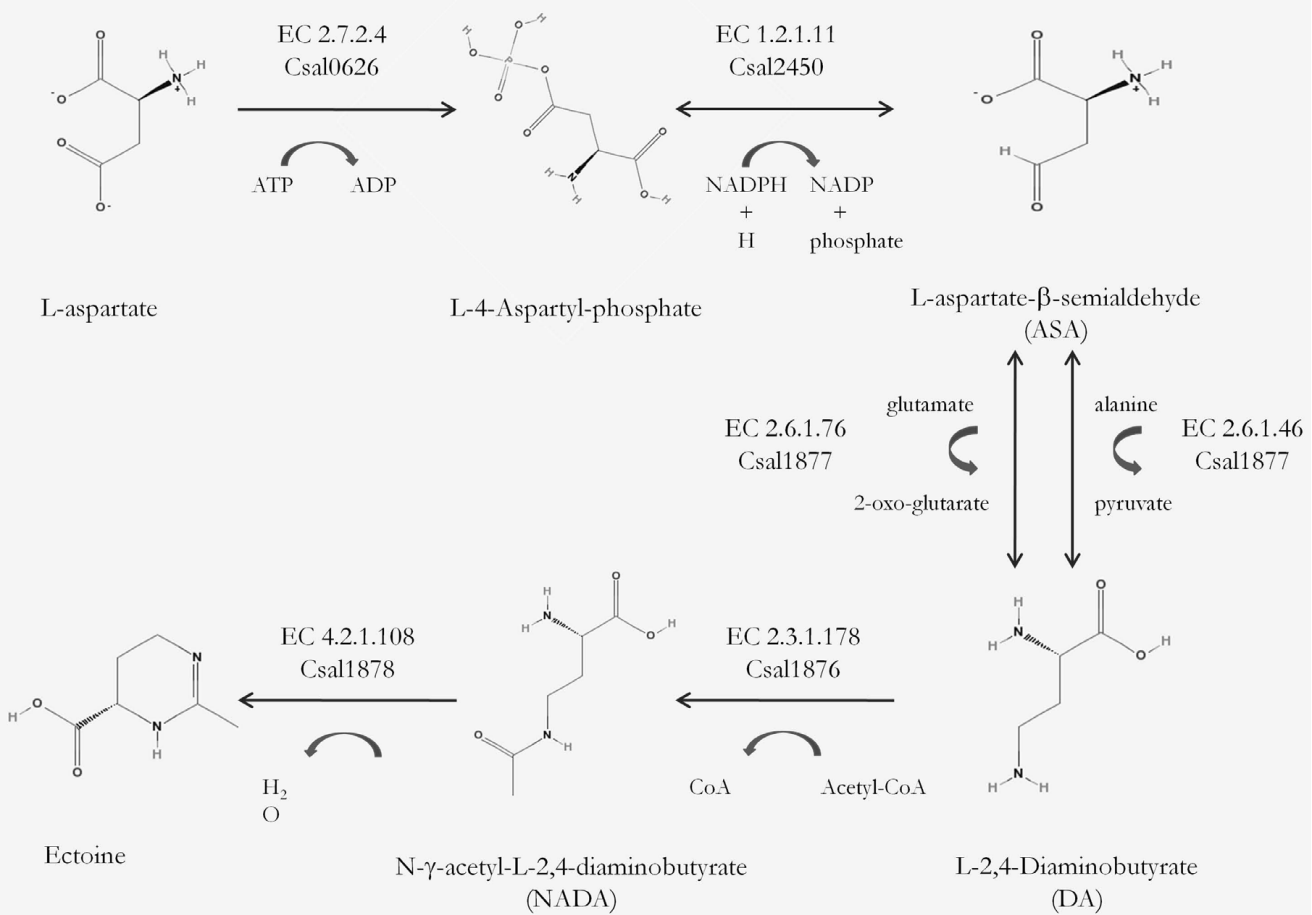
Ectoine biosynthesis pathway in *C. Salexigens*

Due to the high industrial and biological importance of ectoine, current studies are focussed on the elucidation of its biosynthesis mechanism which in turn is essential for the improved production of this compatible solute. Ectoine is synthesized by *C. salexigens *in core osmoadaptation mechanism via ectABC genes (74). Its biosynthesis is a branch of the synthesis pathway for the aspartate family of amino acids (Figure 5). The aspartate is converted into aspartate-β-semialdehyde (ASA) via aspartate kinase (EC 2.7.2.4, Csal0626) and aspartate-semialdehyde dehydrogenase (EC 1.2.1.11, Csal2450), which is further converted to L-2,4-diaminobutyrate (DA) by diaminobutyrate-2-oxoglutarate transaminase (EC 2.6.1.76, Csal1877) requiring glutamate and by diaminobutyrate--pyruvate transaminase (EC 2.6.1.46, Csal1877) in the presence of alanine. L-2,4-diaminobutyrate is acetylated by DA acetyltransferase (EC 2.3.1.178, Csal1876) to Nγ-acetyl-L-2,4-diaminobutyrate (NADA), which is the substrate of ectoine synthase (EC 4.2.1.108, Csal1878).

To simulate metabolic model in the view of ectoine synthesis, the required conditions were implemented and the resulting flux values were investigated. To demonstrate high-level ectoine production when the other external osmoprotectants are not accessible, as stated by Vargas and co-workers (2008); under the absence of exogenous osmoprotectants (i.e. choline and betaine uptake as well as choline oxidation fluxes were constrained to zero), FBA simulations were performed for 3-10 mmol/gDW/h glucose uptake rates. Ectoine production increased from 1.4975 up to 4.9722 mmol/gDW/h with a yield within a range of 49 - 50% mmol ectoine/mmol glucose with concomitant increase in biomass (0.1934 to 0.642 h^-1^) demonstrating the high level ectoine production when glucose was the only carbon source. In addition, Fallet and coworkers (2010) reported batch cultivation data for ectoine production with glucose as the sole carbon source. The performed FBA simulations with 1.5 mmol/gDW/h glucose uptake resulted in an ectoine production rate of 0.75 mmol/gDW/h, which was comparable with the reported experimental result of 0.72 mmol/gDW/h [70].

Comprehensive analysis of the ectoine biosynthesis (Figure 5) revealed the importance of aspartate, glutamate and alanine in directing fluxes through ectoine synthesis pathway. Moreover, key enzymes of the pathway (i.e. aspartate kinase, diaminobutyrate-2-oxoglutarate transaminase, diaminobutyrate--pyruvate transaminase and DA acetyltransferase) link the pathway to the central metabolism. In FBA simulations, the presence of glutamate and alanine in the medium significantly affected both growth and ectoine production. For instance, constraining the glucose and NaCl uptake rates at 1 mmol/gDW/h and 1.1 mmol/gDW/h, respectively; the presence of alanine in the medium was simulated by an uptake rate of 1.2 mmol/gDW/h and the growth was stimulated by 9.01% (from 0.0710 to 0.0774 h^-1^), whereas the ectoine production was improved 9.08% (from 0.5497 to 0.5996 mmol/gDW/h).

## Conclusions

A non-automated but iterative decision-making process was employed in order to reconstruct the first comprehensive genome-scale metabolic model of a halophilic bacterium, *C. salexigens *DSM 3043. The *in silico *model was able not only to represent the potential of the network in terms of phenotypic characterization but also to predict metabolic fluxes during osmoadaptation, both of which were consistent with the experimental observations. The reconstructed model will accelarate the research on halophilic bacteria towards application of systems biology approaches, design of optimal culture conditions and metabolic engineering strategies for improved production of biological and industrially important products.

## Authors' contributions

ETO and KYA conceived and directed the study. OA and KYA designed the algorithms and the reconstruction framework. OA performed the reconstruction process, analyzed the data and evaluated the model. OA, ETO and KYA wrote the paper. All authors read and approved the final version.

## Supplementary Material

Additional file 1 The metabolite and reaction lists of *C*. *salexigens *iOA584 metabolic model (Excel file). Click here for file

Additional file 2 The complete list of genes and their annotations related to the transport mechanisms of *C*. *salexigens *DSM 3043 (Excel file). Click here for file

Additional file 3 Topological analysis of the reconstructed *C*. *salexigens *iOA584 metabolic model (Excel file). Click here for file

Additional file 4 The overall network map and detailed batch images of *C*. *salexigens *iOA584 metabolic model (Word document). Click here for file
