# Supplementary material for: Genome-scale reconstruction of metabolic network for a halophilic extremophile, Chromohalobacter salexigens DSM 3043
Source: BMC Syst Biol. 2011 Jan 21;5:12. doi: 10.1186/1752-0509-5-12 (PMC3034673; doi:10.1186/1752-0509-5-12)
Supplement: Additional file 4 — The overall network map and detailed batch images of C. salexigens iOA584 metabolic model (Word document). [file 1752-0509-5-12-S4.DOC]

Total number of data rows (not including comment lines): 889
Number of rows for which the gene could not be found: 21
Number of rows for which the gene name was ambiguous: 0
Number of rows for which the gene is valid, but for which a data value was missing or malformed: 0

The following table shows statistics for the selected column/column ratio for all genes and for those genes that appear in the Overview (or in any of the selected overviews, if multiple were selected).

| Data Statistics | All Genes | Overview Genes |
| --- | --- | --- |
| Number of values: | 868 | 731 |
| Minimum value: | -1000 | -1000 |
| Maximum value: | 865.7538 | 865.7538 |
| Median: | 4.9724 | 4.9724 |
| Mean: | 37.593365 | 45.910213 |
| Standard deviation: | 225.01498 | 219.16017 |
